# A Cadaveric Study on the Efficacy of Surface Marking and Bony Landmarks Used in Sacral Neuromodulation

**DOI:** 10.7759/cureus.9153

**Published:** 2020-07-12

**Authors:** Sulaiman Almutairi

**Affiliations:** 1 Department of Urology, Majmaah University, Al-Majmaah, SAU

**Keywords:** cadaveric study, observational cross-sectional study, sacral neuromodulation

## Abstract

Background

Anatomical landmarks and surface markings have long been used in out-patient contexts for conducting percutaneous nerve evaluation procedures, but studies testing the reliability of these anatomical landmarks are scant. There have been reports where the procedure has failed. Could it be possible that the anatomical landmarks that are used are not reliable enough? To answer this question, we used this study to understand the reliability of these anatomical landmarks.

Methods

Twenty cadavers, 10 males and 10 females, were dissected in the sacral region; the landmarks were tested, and the angulation and curve made by the sacral 3 (S3) nerve were also studied.

Results

Sacral 3 was identified mainly at the four o’clock position on the right and at the eight o’clock position on the left side. The Sacral 3 foramen was found at a mean distance of 9.17 ± 0.23 cm from the tip of the coccyx. The mean distance of the lateral margin of S3 from the median sacral ridge was found to be 2.16 ± 0.07 cm.

Conclusion

The landmark of 9 cm from the coccyx tip is a valid landmark for sacral neuromodulation (SNM) procedures. The tip of the lead should follow the curve of the nerve as close as possible at the four and eight o’clock positions on the right and left side, respectively. However, the length of the coccyx differs from person to person. The proximity of the adjacent foramina to each other and the variations in the emerging of the nerve are a few factors to be considered while performing SNM procedures. Further study with a larger sample is required in order to investigate the course of the nerve, and its relationship to response to SNM.

## Introduction

Sacral neuromodulation (SNM) is the stimulation of sacral nerves using an implantable neurostimulator for the treatment of various conditions affecting pelvic structures, in order to regulate the functions of urinary and anorectal structures and to enhance sphincteric control for incontinence. Sacral nerves supply pelvic structures, and intervention through neuromodulators helps in the recovery of patients suffering from chronic urinary retention and urge incontinence. In order to locate the sacral nerves during the procedure, knowledge of surface markings with regard to the bony landmarks in the sacral region is necessary. Intervention through sacral neuromodulation is very effective, and it was approved by the FDA in 1997 as a recommended treatment for many conditions involving pelvic structures (e.g., overactive bladder and chronic non-obstructive urine retention) [1]. Various authors have suggested the stimulation of Sacral 3 (S3) in order to produce desirable results, as S3 has minimal influence on the nerve supply of the lower limbs [2]. The stimulation of efferent nerves improves sphincter action, and afferent nerves modulate the reflexes; thus, SNM plays a major role in the recovery of functions in the pelvic structures [1,3]. As stimulation is done with the help of anatomical landmarks, the results of this procedure are highly variable even in the same patient. This is perhaps due to the fact that a proper appreciation of landmarks and their precise location is very challenging. Patients will experience great benefit if the sacral foramina through which the sacral nerves emerge are identified precisely and the electrode is placed appropriately. The improper placement of the needle in the foramina can lead to adverse stimulation of the higherup nerves, and the desired neuromodulation would be difficult to achieve. 

In order to perform this procedure, the greater sciatic notch is palpated; however, this is not easy in many individuals, and some authors, therefore, advocate the use of fluoroscopy to locate and place the lead. However, a careful examination and thorough understanding of the geometry of S3 with respect to the surrounding bony landmarks may reduce dependency on fluoroscopy, as fluoroscopy is somewhat laborious and can be avoided. The questions of how these nerves are identified by surface marking remain. Fluoroscopic procedures have been used and are considered to be very safe procedures. After taking aseptic precautions, the SNM lead is placed in staged implantation or via percutaneous nerve evaluation [4-8]. A high probability of variation between examiners exists, especially in the lumbar spine, including the spinal segments [9-10]. This allows the use of less complicated palpations, like sacroiliac osseous bony landmarks [11]. According to Haas et al., no single study has been done to prove the reliability of the static palpation that is in general use. In order to address this gap, the present study was carried out to describe the geometric parameters and the location of the S3 foramen in relation to various pelvic bony landmarks.

## Materials and methods

Methodology

Study Design

This is an observational cross-sectional study.

*Study Setting* 

This study was conducted at the Department of Anatomy, College of Medicine, Majmaah University.

Study Population

This study included embalmed cadavers from the College of Medicine, Majmaah University, Saudi Arabia.

*Sample Size* 

Twenty embalmed cadavers were used for dissection. Ten were male and ten were female cadavers.

Study Tool

Spinal needles, embalmed cadavers, and standard flexible measuring tape marked to tenths of a centimeter.

*Sampling Technique* 

This study involved convenience sampling.

Data Collection

Before dissection of the sacral region, the distance between the posterior superior iliac spine (PSI) and the tip of the coccyx to the PSI in the midline was recorded. After dissection, internal measurements were also recorded.

Data Analysis

Descriptive statistics are reported as standard deviation (SD), mean, and range. Two-tailed Student’s paired-sample t-tests were done to evaluate the variation. Pearson’s correlation was done for the correlation of various variables.

Ethical Considerations

Ethical clearance was obtained from the Majmaah University ethical board.

The study on SNM therapy was done in the Department of Anatomy, College of Medicine, Majmaah University in Saudi Arabia. The stature, age, and sex of each of the embalmed cadavers were recorded. The distance between the PSI and the tip of the coccyx to the PSI in the midline was documented before dissection. Every cadaver was positioned prone. Before any further assessment, all internal and exterior measurements were taken with a flexible standard measuring tape. For percutaneous lead placement, typically, two spinal needles were placed 2 cm on either side of the midline of the sacrum and 9 cm superior to the coccyx tip. 

A midline incision was taken from the line joining the PSI and the tip of coccyx, and the skin was reflected. A wide-ranging dissection over the sacrum was carried out to reveal the entire sacrum. The needles in the procedure were placed leftward throughout the dissection so that the site of each needle tip in connection to the upper part of the 3rd sacral foramen could be assessed. Each foramen is identifiable if the bone is visible. For the upper part of the 3rd sacral foramen, the distance from the tip of each needle was assessed. It was also noted if the needle entered the foramen. From the tip of the coccyx to the level of S3, bilaterally, all of the dimensions were measured, as well as from the middle ridge of the sacrum of the S3 lateral aspect to the foramina on either side. Measurements between the lower border of Sacral 2 and the upper border of 3rd sacral foramen, and the lower border of S3 and the upper border of S4 were taken on either side, so as to obtain the inter-foramina distance.

## Results

Table 1 shows the various morphometric measurements of sacrum in male and female cadavers.

**Table 1:** Description of various morphometric measurements of sacrum in male and female cadavers WC, wrist circumference; IP-E, inter-posterior superior iliac spine length (external); IP-I, inter-posterior superior iliac spine length (internal); C-PSI E, coccyx to a level of PSI (external); C-PSI I, coccyx to a level of PSI (internal); N-S-S3, needle tip to the superior aspect of S3; TC-S-S3, tip of the coccyx to the superior aspect of S3; MSR-LS3, middle sacral ridge to the lateral aspect of S3; IMS2-SMS3, inferior margin of S2 to the superior margin of S3; IMS3-SMS4, inferior margin of S3 to the superior margin of S4; SIJ-SSMS3, inferior level of SI joint to superior margin of S3; SIJ-SSMS2, inferior level SI joint to superior margin of S2

Morphometric Variables			Mean ± SD in (cms)	Median (IQR) in (cms)	Range in (cms)
Female	Height	10	157.54 ± 4.53	156.9 (155.7, 161.7)	149.3–163.3
	W C	10	16.13 ± 0.61	16.25 (15.8, 16.6)	15–16.9
	IP-E	10	9.45 ± 0.23	9.5 (9.2, 9.6)	9.1–9.8
	IP-I	10	8.9 ± 0.27	9.05 (8.6, 9.1)	8.5–9.2
	C-PSI E	10	11.6 ± 0.2	11.6 (11.4, 11.8)	11.3–11.9
	C-PSI I	10	12.2 ± 0.2	12.3 (12.1, 12.4)	11.9–12.5
	N-S-S3	10	1.34 ± 0.07	1.35 (1.3, 1.4)	1.2–1.4
	TC-S-S3	10	9.03 ± 0.16	9 (8.9, 9.2)	8.8–9.3
	MSR-LS3	10	2.16 ± 0.08	2.2 (2.1, 2.2)	2–2.3
	IMS2-SMS3	10	1.39 ± 0.07	1.4 (1.3, 1.4)	1.3–1.5
	IMS3-SMS4	10	1.39 ± 0.06	1.4 (1.4, 1.4)	1.3–1.5
	SIJ-SSMS3 Right	10	0.77 ± 0.05	0.8 (0.7, 0.8)	0.7–0.8
	SIJ-SSMS3 Left	10	0.77 ± 0.05	0.8 (0.7, 0.8)	0.7–0.8
	SIJ-SSMS2 Right	10	1.83 ± 0.09	1.8 (1.8, 1.9)	1.7–2
	SIJ-SSMS2 Left	10	1.83 ± 0.09	1.8 (1.8, 1.9)	1.7–2
	Angle S3 Right	10	80.9 ± 4.58	79 (78, 82)	77–90
	Angle S3 Left	10	79.5 ± 4.74	78.5 (77, 84)	71–87
Male	Height	10	171.67 ± 2.97	171.4 (169.7, 172.7)	167.2–177.3
	WC	10	17.46 ± 0.6	17.35 (17.1, 17.8)	16.5–18.6
	IP-E	10	8.51 ± 0.25	8.5 (8.3, 8.7)	8.2–8.9
	IP-I	10	8.09 ± 0.22	8.1 (7.9, 8.3)	7.8–8.4
	C-PSI E	10	13.1 ± 0.1	13.1 (13.1, 13.2)	12.9–13.4
	C-PSI I	10	13.5 ± 0.2	13.5 (13.4, 13.6)	13.3–13.9
	N-S-S3	10	1.11 ± 0.07	1.1 (1.1, 1.2)	1–1.2
	TC-S-S3	10	9.3 ± 0.21	9.25 (9.1, 9.4)	9.1–9.7
	MSR-LS3	10	2.15 ± 0.05	2.15 (2.1, 2.2)	2.1–2.2
	IMS2-SMS3	10	1.51 ± 0.09	1.5 (1.5, 1.5)	1.4–1.7
	IMS3-SMS4	10	1.5 ± 0.07	1.5 (1.5, 1.5)	1.4–1.6
	SIJ-SSMS3 Right	10	0.77 ± 0.05	0.8 (0.7, 0.8)	0.7–0.8
	SIJ-SSMS3 Left	10	0.77 ± 0.05	0.8 (0.7, 0.8)	0.7–0.8
	SIJ-SSMS2 Right	10	2.07 ± 0.05	2.1 (2, 2.1)	2–2.1
	SIJ-SSMS2 Left	10	2.07 ± 0.05	2.1 (2, 2.1)	2–2.1
	angle S3 right	10	80.4 ± 3.89	78.5 (78, 83)	77–89
	angle S3 left	10	80.9 ± 2.85	81 (78, 83)	77–86

In our study, the average height after calculation of the subjects was found to be 164.61 cm. Male cadavers were found to be taller when compared to the female. The average wrist circumference, which is also a good indicator in the field of physical anthropology, was found to be more in males (16.79 cm) than females cadavers (16.13 cm).

The mean distance between the posterior superior iliac spine length (externally measured and they are the bony landmarks that are felt on the back) was found to be 8.98 cm. In males, it was found to be less than females. The same when measured after dissection was found to be 8.5±0.48 cm and was noted to be more in males than females.

The average distance between the Coccyx (tail bone) and the level of the line joining the previous landmarks was measured to be 12.37±0.79 cm. After dissection, the results were similar. The average distance between the needle tip and the superior aspect was 1.23±0.14 cm. 

The mean IP-E before dissection was 8.51 cm and 9.45 cm in male and female cadavers and was significantly more than mean IP-I taken after dissection (males: 8.09 cm and females: 8.9 cm). However, the measurement C-PSI was noted to be more after dissection than the measurement taken before dissection. C-PSI-E in males and females was 13.12 cm and 11.61 cm, respectively. On the contrary, C-PSI- I measured 13.52 cm and 12.22 cm in males and females, respectively. The tip of coccyx which is the most inferior part of the tailbone to the superior aspect of third sacral foramen was found to be 9.17 cm. In males, it was a bit more when compared to that of the female. The middle sacral ridge that is the bony plate of bone that forms a divider in between the sacrum and divides it into right and left parts to the lateral aspect of the 3rd sacral foramen was found to be 2.16±0.07 cm. In males and females, the difference was very negligible. The mean angle of S3 (3rd sacral nerve) on the right was found to 80.65±4.15 degrees. On the left, it was found to be 80.2±3.87 degrees.

A positive correlation was observed between various parameters like height and waist circumference, height and TC-S-S3, C-PSI E & TC-S-S3, C-PSI I & TC-S-S3, WC & TC-S-S3, and WC & TC-S-S3. Likewise, a negative correlation was noted in parameters like IP-E & TC-S-S3, IP-I & TC-S-S3, and IP-I & C-PSI I. 

**Figure 1:**
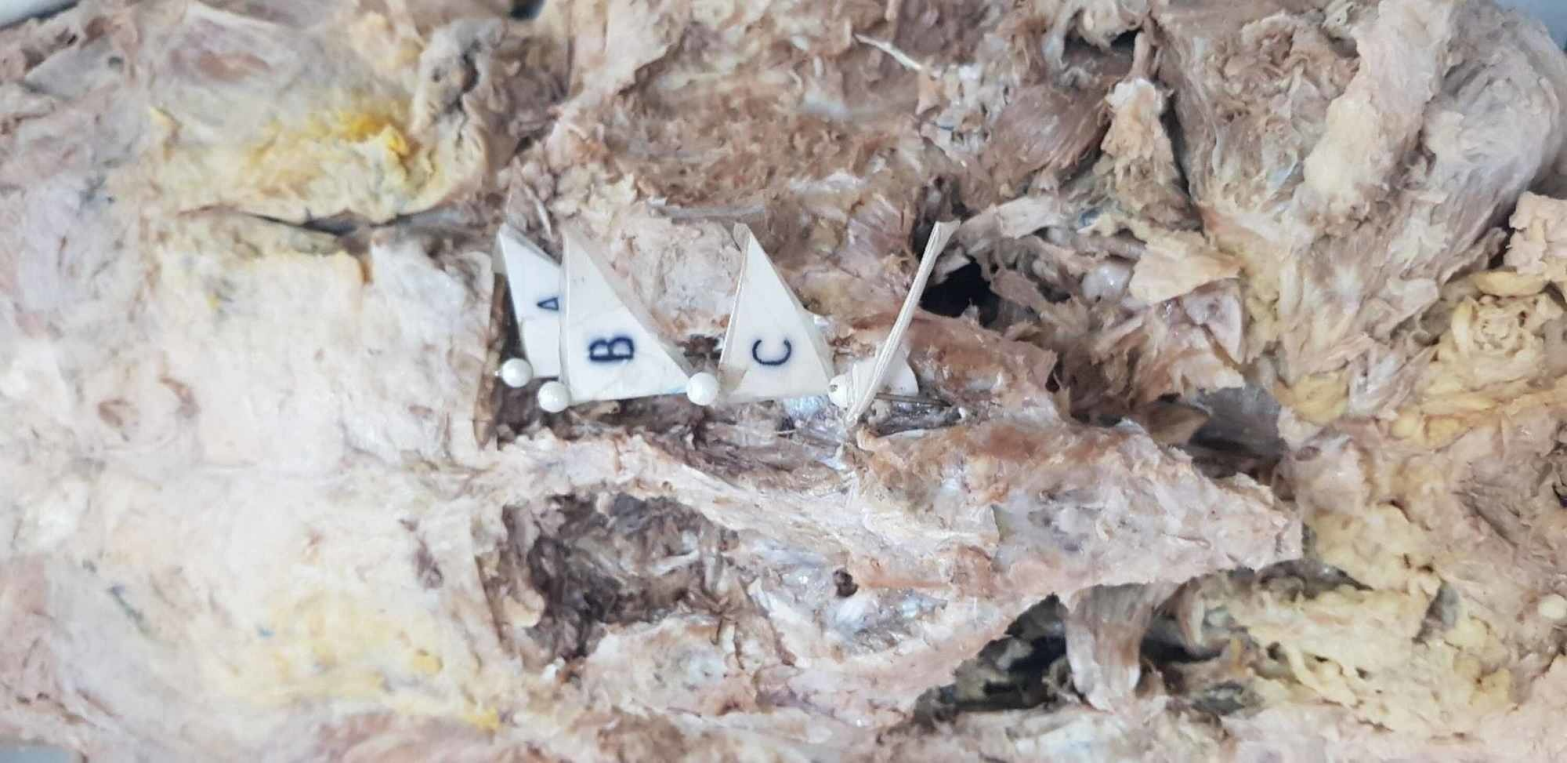
Dissection of the sacral region with flagged foramen A: First sacral foramen right side, B: Second sacral foramen right side, C: Third sacral foramen right side, D: Fourth sacral foramen right side. On dissection of the sacrum, the foramen were exposed, the sacral curvature and the angel of the inlet were noted down. The distance between midline and coccygeus was measured.

Figure 1 shows a dissection of the sacral region with flagged foramen.

A positive correlation means that as one parameter value increases, the other also increases. A negative correlation means that as one parameter increases, the other decreases.

The parameters of height and waist circumference (WC) showed a good significant positive correlation with a p-value of 0.002. The parameters of height and tip of the coccyx to the superior aspect of S3 (TC-S-S3) showed an excellent significant positive correlation with a p-value of <0.001. The parameters of inter-posterior superior iliac spine length (external) (IP-E) and TC-S-S3 showed a good significant negative correlation with a p-value of 0.021. The parameters of IP-I and TC-S-S3 showed a good significant negative correlation with a p-value of 0.021. The parameters of coccyx to a level of PSI (external) (C-PSI E) and TC-S-S3 showed a very good significant positive correlation with a p-value of <0.001. The parameters of C-PSI I and TC-S-S3 showed a good significant positive correlation with a p-value of <0.001. The parameters of IP-I and C-PSI I showed an excellent significant negative correlation with a p-value of <0.001. The parameters of WC and TC-S-S3 showed a good significant positive correlation with a p-value of 0.036 (Table 2).

**Table 2:** Pearson’s correlation for correlation of various variables WC, wrist circumference; IP-E, inter-posterior superior iliac spine length (external); IP-I, inter-posterior superior iliac spine length (internal); C-PSI E, coccyx to a level of PSI (external); C-PSI I, coccyx to a level of PSI (internal); TC-S-S3, tip of the coccyx to the superior aspect of S3; MSR-LS3, middle sacral ridge to the lateral aspect of S3

SNO	Parameters being Correlated	N	Correlation (r)	p-Value
1	Height and WC	20	0.643	0.002
2	Height and TC-S-S3	20	0.834	<0.001
3	IP-E and TC-S-S3	20	−0.511	0.021
4	IP-I and TC-S-S3	20	−0.511	0.021
5	C-PSI E and TC-S-S3	20	0.722	<0.001
6	C-PSI I and TC-S-S3	20	0.718	<0.001
7	IP-I and C-PSI I	20	−0.807	<0.001
8	WC and TC-S-S3	20	0.471	0.036

A comparison of the height and WC between the two groups showed that height and WC of males were found to be more than females (P value<0.05). A comparison of the IP-E between the two groups showed that IP-E and IP-I were higher in the F group and were statistically significant with a p-value of <0.001. 

It was noted that the male group had higher C-PSI E and C-PSI I when compared to females (P-value <0.01). The N-S-S3 distance was found to be more in females than in males with a p-value of <0.001. A comparison of the TC-S-S3 between the two groups showed that TC-S-S3 was higher in the M group P-value of 0.005. On comparing the IMS2-SMS3 between the two groups showed that IMS2-SMS3 was higher in the M group was statistically significant with a P-value of 0.004. 

Further, the measurements after dissection showed that IMS3-SMS4, SIJ-SSMS3, SIJ-SSMS2 right, and SIJ-SSMS2 left were considerably on a higher side in males as compared to females with an exception of angle S3 right measurement higher in females with a P-value of <0.05. (Table 3).

**Table 3:** Comparison of various measurements between gender WC, wrist circumference; IP-E, inter-posterior superior iliac spine length (External); IP-I, inter-posterior superior iliac spine length (Internal); C-PSI E, coccyx to a level of PSI (External); C-PSI I, coccyx to a level of PSI (Internal); N-S-S3, needle tip to the superior aspect of S3; TC-S-S3, tip of the coccyx to the superior aspect of S3; MSR-LS3, middle sacral ridge to the lateral aspect of S3; IMS2-SMS3, inferior margin of S2 to the superior margin of S3; IMS3-SMS4, inferior margin of S3 to the superior margin of S4; SIJ-SSMS3, inferior level of SI joint to superior margin of S3; SIJ-SSMS2, inferior level SI joint to superior margin of S2

	M (n = 10)	F (n = 10)	p-Value
	Mean ± SD	Mean ± SD	
Height	171.67 ± 2.97	157.54 ± 4.53	<0.001
WC	17.46 ± 0.6	16.13 ± 0.61	<0.001
IP-E	8.51 ± 0.25	9.45 ± 0.23	<0.001
IP-I	8.09 ± 0.22	8.9 ± 0.27	<0.001
C-PSI E	13.12 ± 0.13	11.61 ± 0.22	<0.001
C-PSI I	13.53 ± 0.17	12.22 ± 0.22	<0.001
N-S-S3	1.11 ± 0.07	1.34 ± 0.07	<0.001
TC-S-S3	9.3 ± 0.21	9.03 ± 0.16	0.005
MSR-LS3	2.15 ± 0.05	2.16 ± 0.08	0.754
IMS2-SMS3	1.51 ± 0.09	1.39 ± 0.07	0.004
IMS3-SMS4	1.5 ± 0.07	1.39 ± 0.06	0.001
SIJ-SSMS3 Right	0.77 ± 0.05	0.77 ± 0.05	1
SIJ-SSMS3 Left	0.77 ± 0.05	0.77 ± 0.05	1
SIJ-SSMS2 Right	2.07 ± 0.05	1.83 ± 0.09	<0.001
SIJ-SSMS2 Left	2.07 ± 0.05	1.83 ± 0.09	<0.001
Angle S3 Right	80.4 ± 3.89	80.9 ± 4.58	0.796
Angle S3 Left	80.9 ± 2.85	79.5 ± 4.74	0.434

It was found that the S3 nerve emerged on the right side at a four o’clock position in eight cases and at a three o’clock position in two cases. On the left side, an eight o’clock position was observed in seven cases and a three o’clock position was observed in three cases (Table 4).

**Table 4:** S3 nerve outlet in terms of position

	4 o’clock	3 o’clock	8 o’clock	9 o’clock
Right	8	2		
Left			7	3

## Discussion

Sacral nerve stimulation is used for several varying pathologies that range from those concerned with the urinary bladder to those concerned with fecal inconsistencies, as well as in the field of obstetrics and gynecology [12-13]. The procedure is known not only to improve the symptoms in patients but also to improve quality of life [14-16]. Neuromodulation can be practiced in any of the sacral nerves, but the lower ones are more preferred, as the incidence of the nerve root and vascular structures greatly increases in the higher sacral nerves when compared to the lower ones [17]. A procedure at the level of S3 produces the best motor response [18]. In a study conducted by Hasan *et al*. [17], it was reported that in the second sacral foramen, the nerve occupies almost half of the foramen, thereby increasing the chances of injury. The third and the fourth sacral nerves occupy less than a quarter of the foramen. This is ideal for the procedure.

In a study conducted by Deveneau *et al*. [19], the mean height of the subjects was found to be 163.7 cm with a standard deviation of ±10.3 cm. In male cadavers, it was found to be 170.8 cm, with a standard deviation of ±5.2 cm. In female cadavers, it was found to be 156.7 cm, with a standard deviation of ±9.3 cm. The wrist circumference is considered as an important measurement in physical anthropology and is related to various sacral morphometric measurements. The mean wrist circumference of the subjects was found to be 17.00 cm, with a standard deviation of ±1.2 cm. In male cadavers, it was found to be 17.7 cm, with a standard deviation of ±0.9 cm. In female cadavers, it was found to be 16.3 cm, with a standard deviation of ±1.2 cm.

In the study conducted by Deveneau *et al*. [19], a mean needle tip to a superior aspect of S3 was measured to be 1.2 ± 1.3 cm. In males, it was found to be 1.1 ± 1.1 cm, and in females, it was found to be 1.4 ± 1.5 cm, and the mean tip of the coccyx to the superior aspect of S3 was found to be 9.3 ± 0.9 cm. In males, it was found to be 9.4 ± 1.0 cm, and in females, it was found to be 9.2 ± 0.7 cm. The middle sacral ridge to the lateral aspect of S3 was found to be 2.3 ± 0.2 cm. In males, it was found to be 2.3 ± 0.2 cm, and in females, it was found to be 2.3 ± 0.2 cm. These measurements and their differences help in accurately placing the needles in the canal by a practicing urologist. If not followed, it may lead to an unnecessary stimulation of higher up nerves which can have some extra innervations. 

To know the actual distance between S2 and S3 foramen is important because of their proximity and any increase in the angle of insertion of the needle may lead to adverse stimulation. The mean inferior margin of S2 to the superior margin of S3 was found to be 1.5 ± 0.3 cm. In males, it was found to be 1.6 ± 1.6 cm. In females, it was found to be 1.4 ± 0.2 cm. In the study conducted by Hasan *et al.* [17], the inter-foraminal distance between S2 and S3 was found to be 3 ± 0.1 cm in males and 2 ± 0.1 cm in females [19]. The mean inferior margin of S3 to the superior margin of S4 was found to be 1.5 ± 0.2. In males, it was found to be 1.5 ± 0.5 cm. In females, it was found to be 1.4 ± 0.2 cm.

Hasan ST *et al*. [17] showed that the inter-foraminal distance between S3 and S4 was found to be 3 ± 0.1 cm in males and 2 ± 0.1 cm in females.

In the study conducted by Deveneau *et al*. [19], the mean inferior level of the SI joint to the superior margin of S3 on the right side was found to be 0.8 ± 0.5 cm. On the left side, it was found to be 0.7 ± 0.4 cm. It was found that the S3 nerve emerged out on the right side at a four o’clock position in eight cases and in a three o’clock position in two cases. On the left side, an eight o’clock position was observed in seven cases and a three o’clock position was observed in three cases.

In a study conducted by Hasan *et al*. [17], in males, the angles made by S3 were observed to be 103 ± 5 (90-110) degrees in males, and 106 ± 13 (72-118) degrees in females. Hence, the needle was introduced at the same angle to attain the nerve stimulation.

In a study conducted by Buchs *et al*. [2], the left side angle at S3 was noted to be 91.7 ± 13.5 (80-110) degrees. In males, it was found to be 80.3 ± 0.6 (80-81) degrees, and in females, it was observed to be 103 ± 8.2 (94-110) degrees. On the right side, 83.2 ± 7.7 (75-95) degrees were observed and in males, 87.5 ± 10.6 (80-95) degrees were observed, and on the left side, 80.3 ± 5.5 (75-86) degrees were observed.

In terms of the identification of external measurements that correlate with internal sacral parameters, there was no relationship found between them. In our study, we found that the correlation between the parameters of height and TC-S-S3 showed a positive significant correlation, with a p-value of <0.001.

Although it is a fair estimate, one has to understand the fact that the length of the coccyx differs from person to person. The proximity of the adjacent foramina to each other and the variations in the emerging of the nerve are a few determinants that need to be remembered while performing the sacral neuromodulation procedure. The limitations of this study include a small sample size, single-center study, single-examiner dissections, and embalmed cadavers which are not as reliable as fresh cadavers. Future research should include a larger sample size from multiple centers and examinations on fresh cadavers so as to assess intra- and inter-examiner reliability.

## Conclusions

The landmark of 9 cm from the coccyx tip is a valid landmark for SNM procedures. The tip of the lead should follow the curve of the nerve as close as possible to the four and eight o’clock position in the right and left side, respectively. However, the length of the coccyx differs from person to person. The proximity of the adjacent foramina to each other, and the variations in the emerging of the nerve are a few decisional considerations that need to be balanced while performing these procedures. Further studies with a larger sample are required to investigate the course of this nerve and its relationship to response to SNM.

## Appendices

**Table 5:** Appendix A WC, wrist circumference; IP-E, inter-posterior superior iliac spine length (External); IP-I, inter-posterior superior iliac spine length (Internal); C-PSI E, coccyx to a level of PSI (External); C-PSI I, coccyx to a level of PSI (Internal); N-S-S3, needle tip to the superior aspect of S3; TC-S-S3, tip of the coccyx to the superior aspect of S3; MSR-LS3, middle sacral ridge to the lateral aspect of S3; IMS2-SMS3, inferior margin of S2 to the superior margin of S3; IMS3-SMS4, inferior margin of S3 to the superior margin of S4; SIJ-SSMS3, inferior level of SI joint to superior margin of S3; SIJ-SSMS2, inferior level SI joint to superior margin of S2

Sl No	Sex	Height	WC	IP-E	IP-I	C-PSI E	C-PSI I	N-S-S3	TC-S-S3	MSR-LS3	IMS2-SMS3	IMS3-SMS4	SIJ-SSMS3	SIJ-SSMS3	SIJ-SSMS2	SIJ-SSMS2	Angle S3	Angle S3	Angle S3	Angle S3	Sl No
													Right	Left	Right	Left	Right	Left	Right	Left	
1	M	171.4	17.8	8.7	8.2	13.1	13.5	1.1	9.6	2.2	1.5	1.5	0.8	0.8	2.1	2.1	83	81	4 o’clock	8 o’clock	1
2	M	177.3	17.1	8.3	7.8	13.4	13.9	1.2	9.7	2.1	1.6	1.6	0.8	0.8	2	2	84	83	4 o’clock	8 o’clock	2
3	M	167.2	17.3	8.2	7.8	12.9	13.3	1.1	9.1	2.1	1.4	1.5	0.7	0.7	2.1	2.1	79	78	4 o’clock	8 o’clock	3
4	M	169	16.5	8.4	8	13	13.4	1	9.1	2.1	1.5	1.4	0.8	0.8	2	2	81	84	4 o’clock	8 o’clock	4
5	M	171.4	16.9	8.4	7.9	13.1	13.5	1.1	9.2	2.2	1.5	1.5	0.7	0.7	2	2	77	81	4 o’clock	8 o’clock	5
6	M	170.3	18.6	8.9	8.4	13.1	13.5	1.2	9.2	2.2	1.5	1.5	0.8	0.8	2.1	2.1	78	77	4 o’clock	8 o’clock	6
7	M	175.3	18.1	8.6	8.2	13.2	13.7	1.1	9.4	2.2	1.7	1.6	0.8	0.8	2.1	2.1	78	78	4 o’clock	8 o’clock	7
8	M	172.7	17.4	8.8	8.3	13.1	13.5	1	9.3	2.2	1.5	1.5	0.7	0.7	2.1	2.1	77	80	4 o’clock	8 o’clock	8
9	M	172.4	17.3	8.2	8	13.2	13.6	1.1	9.3	2.1	1.5	1.4	0.8	0.8	2.1	2.1	78	81	4 o’clock	8 o’clock	9
10	M	169.7	17.6	8.6	8.3	13.1	13.4	1.2	9.1	2.1	1.4	1.5	0.8	0.8	2.1	2.1	89	86	3 o’clock	9 o’clock	10
11	F	156.3	15.8	9.6	9.1	11.7	12.1	1.4	8.9	2.2	1.3	1.4	0.8	0.8	1.9	1.9	88	87	3 o’clock	9 o’clock	11
12	F	161.7	15	9.4	9	11.8	12.2	1.3	9.1	2.1	1.4	1.4	0.8	0.8	2	2	81	77	4 o’clock	8 o’clock	12
13	F	157.4	15.9	9.7	9.2	11.5	12.1	1.3	9	2.2	1.4	1.4	0.7	0.7	1.8	1.8	78	76	4 o’clock	8 o’clock	13
14	F	159.5	15.4	9.5	9.1	11.7	12.3	1.2	9.2	2.2	1.4	1.4	0.8	0.8	1.8	1.8	90	85	4 o’clock	9 o’clock	14
15	F	163.3	16.6	9.5	9.1	11.9	12.5	1.4	9.3	2.1	1.5	1.5	0.7	0.7	1.9	1.9	79	71	4 o’clock	8 o’clock	15
16	F	149.3	16.8	9.2	8.8	11.3	12.3	1.3	8.8	2.3	1.3	1.4	0.8	0.8	1.7	1.7	77	78	4 o’clock	8 o’clock	16
17	F	155.7	16.2	9.8	9.1	11.4	12.4	1.3	8.9	2	1.4	1.3	0.8	0.8	1.8	1.8	78	79	4 o’clock	8 o’clock	17
18	F	163.2	16.9	9.5	8.6	11.9	12.5	1.4	9.2	2.1	1.5	1.4	0.7	0.7	1.9	1.9	77	78	4 o’clock	8 o’clock	18
19	F	152.6	16.4	9.2	8.5	11.4	11.9	1.4	8.9	2.2	1.3	1.3	0.8	0.8	1.7	1.7	79	80	4 o’clock	8 o’clock	19
20	F	156.4	16.3	9.1	8.5	11.5	11.9	1.4	9	2.2	1.4	1.4	0.8	0.8	1.8	1.8	82	84	4 o’clock	8 o’clock	20
